# Timing of Delivery of Low‐Risk Persons and the Risk of Attention‐Deficit Hyperactivity Disorder in Offspring: Sweden and British Columbia, Canada

**DOI:** 10.1111/ppe.13162

**Published:** 2025-01-07

**Authors:** Thi Hoang Ha Nguyen, M. Zakir Hossin, Stefanie Schmauder, Giulia M. Muraca, Sarka Lisonkova, Neda Razaz

**Affiliations:** ^1^ Clinical Epidemiology Division, Department of Medicine Solna Karolinska Institutet Stockholm Sweden; ^2^ School of Population and Public Health University of British Columbia Vancouver British Columbia Canada; ^3^ Department of Obstetrics and Gynecology, Faculty of Health Sciences McMaster University Hamilton Ontario Canada; ^4^ Department of Health Research Methods, Evidence, and Impact, Faculty of Health Sciences McMaster University Hamilton Ontario Canada; ^5^ Department of Obstetrics and Gynaecology, BC Children's and Women's Hospital and Health Centre The University of British Columbia Vancouver British Columbia Canada

**Keywords:** ADHD, low‐risk pregnancies, neurodevelopmental disorders, timing of delivery

## Abstract

**Background:**

An evidence gap exists concerning the timing of delivery at 37–42 weeks and the risk of attention‐deficit hyperactivity disorder (ADHD) in offspring.

**Objective:**

To determine the association between timing of delivery in low‐risk pregnancies at term (37–42 weeks) gestations and ADHD in offspring.

**Methods:**

This population‐based cohort study comprised 1,424,453 singletons in Sweden and 403,765 in British Columbia (BC), Canada, live‐born at 37–42 completed weeks to low‐risk pregnant women between 2000 and 2018. Children were followed up from age 1 until the date of death, emigration, their first diagnosis, or December 2020 (study's end date). The exposure was time of delivery assessed through gestational age, and the outcome was the diagnosis of ADHD. Cox regression models were used to examine the association between gestational age at delivery and ADHD.

**Results:**

During the follow‐up period, 59,989 children in Sweden were diagnosed with ADHD (4.5 per 1000 child‐years). Correspondingly, in BC, during the same period, there were 27,445 children diagnosed with ADHD (7.4 per 1000 child‐years). In Sweden, the adjusted hazard of ADHD was 10%, 6%, and 3% higher at 37, 38, and 39 weeks gestation compared with those born at ≥ 38, ≥ 39, and ≥ 40 weeks, respectively. In BC, the corresponding hazards were 9%, 6%, and 3%, respectively. Both regions showed no elevated ADHD risks for infants born at 40 weeks compared to those born at ≥ 41 weeks, with slightly lower rates at 40 weeks.

**Conclusions:**

In low‐risk pregnancies, births at 37 and 38 weeks were associated with a higher ADHD risk, while births at 40 weeks showed no increased risk compared with those born at later gestations.

## 1 Background

In uncomplicated singleton pregnancies, induction of labour is generally recommended from 41 + 0 weeks' gestation [1, 2]. In many centres in the UK and Scandinavian countries, it is common practice to perform induction by the 42nd week of pregnancy. Recent studies suggest that induction at 39 weeks [3] or 41 weeks gestation [4] results in a reduction in caesarean rates, perinatal mortality and adverse neonatal outcomes compared with expected management.

Systematic reviews and meta‐analyses present mixed findings regarding neonatal outcomes following induction of labour versus expectant management in uncomplicated, singleton and term pregnancies. A study involving over 650,000 low‐risk nulliparous women showed that elective labour induction at 39 weeks decreased the risk of respiratory morbidity, neonatal intensive care unit (NICU) admission and mortality [5]. Conversely, another meta‐analysis of five randomised controlled trials (RCTs) found no difference in admission to the NICU and neonatal mortality but noted a reduction in required respiratory support in the induction arm [6]. One meta‐analysis of RCTs comparing induction at 41 weeks versus expectant management until 42 weeks indicated reduced mortality and NICU admissions [7]. Additionally, a Cochrane review of 34 RCTs suggested that induction of labour at 37 to 41 weeks reduced the risk of NICU admission and the risk of a 5‐min Apgar score below 7 [8].

Preterm infants are at elevated risk of adverse neurodevelopment outcomes [9]. However, among low‐risk pregnancies at term gestation, a key knowledge gap in obstetric management is the lack of data on long‐term neurodevelopmental outcomes [8, 10]. A recent population‐based study of 1.8 million low‐risk pregnancies in Sweden showed that being born at 39 and 40 weeks, compared with later gestation, reduced the risk of stillbirth, infant mortality, and cerebral palsy [11]. This study supports the findings that 39 + 0 to 40 + 6 weeks might be the optimal time for delivery for low‐risk pregnancies. However, there is little evidence to show whether the timing of delivery is associated with other neurodevelopmental outcomes [8]. Therefore, this population‐based study of over 1.8 million children born in Sweden and British Columbia (BC), Canada, aims to determine the association between timing of delivery and risk of ADHD in singleton births at 37–42 completed weeks among low‐risk pregnancies.

## 2 Methods

### 2.1 Data Sources

This study utilised data from the Swedish Medical Birth Register [12] and the British Columbia Vital Statistics Birth File [13], covering over 98% of all births in Sweden and BC, Canada. The Swedish Medical Birth Register provides comprehensive information on pre‐pregnancy, perinatal and neonatal characteristics from antenatal and delivery medical records. In Sweden, this register was linked with several national databases using a unique 10‐digit personal identification, including the National Patient Register (for hospitalisation and outpatient care) [14], the Prescribed Drug Register [15], the Cause of Death Registers [16] and the Total Population Register [17]. In BC, the Perinatal Database Registry [18] was linked with the Discharge Abstract Database (for hospitalizations) [19], the Medical Services Plan physician billing data (for outpatient physician visits) [20], PharmaNet (for drug exposure) [21], the British Columbia Vital Statistics Death File [22] and the Central Demographics File (for detailed demographic information) [23].

### 2.2 Study Population

Between 1 January 2000 and 31 December 2018, we identified 2,006,356 singleton live births from the Swedish Medical Birth Register and 776,775 births from the BC Perinatal database. We excluded infants with missing maternal or child identification numbers, those born at less than 37 or ≥ 43 + 0 weeks, those with any malformation during the first year (identified by International Classification of Disease [ICD]‐9 codes: 740–759, ICD‐10 codes: Q00‐Q99) and those who died or emigrated before reaching 1 year of age. Since our focus was on low‐risk pregnancies, we further excluded infants of mothers who had a breech presentation, chronic diseases and selected pregnancy complications. All ICD‐10 codes used to identify these and other conditions are shown in Table S1. The final study population included 1,424,453 children in Sweden (Figure S1) and 403,765 children in BC (Figure S2).

### 2.3 Exposures

Gestational age (in completed weeks) was estimated following a hierarchical approach depending on the availability of information: the date of early second‐trimester ultrasound (90.5%), the date of the last menstrual period (5.4%) and a post‐natal assessment (4.1%). To assess outcomes at each gestational week, we compared deliveries at a given gestational week with those at later gestational weeks, representing expectant management. This comparison group included all women who delivered at a later gestational age by any onset of labour or mode of delivery. This comparison is more clinically relevant and better suited to guide clinical decision‐making. For instance, the impact of delivery at 37 weeks was assessed by comparing outcome rates between births at 37 + 0 to 37 + 6 weeks versus births at 38 + 0 to 42 + 6 weeks.

### 2.4 Outcomes

Individuals with ADHD were identified as those who, at age 1 or older, had either an ADHD diagnosis recorded in the National Patient Register in Sweden or the Discharge Abstract Database and Medical Services Plan in the BC cohort or who received their first prescription for ADHD medication. ADHD medication prescriptions were identified using Anatomical Therapeutic Chemical (ATC) codes from the Prescribed Drug Register in Sweden (available since July 2005) and from PharmaNet in BC (available since 1996). All ICD‐10 and ATC codes used in the analyses are shown in Table S2. ICD‐9 codes were used for disease identification until 1997 in Sweden and 2001 in BC, after which ICD‐10 coding standards were adopted. The date associated with the first recorded diagnosis of ADHD was considered the date of diagnosis.

### 2.5 Other Variables

Maternal characteristics examined were age at delivery, country of birth, education level, smoking during pregnancy, marital status/cohabitation with partner, parity and height. Mothers who reported daily smoking at the first antenatal visit or 30 to 32 gestational weeks were classified as smokers (Sweden only). Information on the onset of labour (spontaneous, induced or caesarean delivery) was obtained from obstetric records, and the year of delivery was examined to address temporal changes in obstetrical practice. We also captured both parents' history of any neurodevelopmental and psychiatric disorders before the child's date of birth, using inpatient and outpatient data (Table S2).

### 2.6 Statistical Analyses

The distributions of infant and parental characteristics were quantified for births occurring at each gestational week. We calculated the incidence rates per 1000 person‐years with 95% confidence intervals (CI) for ADHD according to gestational age at birth. For each outcome, children were followed from 1 year of age until the date of diagnosis of the outcome, death, emigration or the end of the study on 31 December 2020, whichever came first. Hazard ratios (HR) and 95% CI were calculated using Cox proportional hazards regression models to estimate the associations between gestational age and the outcomes. Child's attained age was used as the underlying time metric in all Cox models. Estimates in the multivariable analyses were adjusted for maternal factors (maternal age, height, country of birth, parity, education level, smoking and mother's and father's history of neurodevelopmental disorders), child's sex and year of delivery. We also performed stratified analyses by the onset of planned caesarean delivery (no labour, spontaneous and induced). In this analysis, the rates of outcomes at any gestational week within each onset of labour category were compared with the rates of outcome among all births at subsequent gestational weeks, regardless of labour onset. We conducted separate analyses for the Sweden and BC cohorts.

### 2.7 Missing Data

Since some covariates, such as maternal body mass index (BMI), had missing values (ranging from 0.1% for country of birth in Sweden to 26% for maternal BMI in BC), we imputed missing data through multiple imputations using a chained equations approach. We assumed that the pattern of missing data was ‘missing at random’ and created 50 imputed datasets (after 200 burn‐in iterations).

### 2.8 Sensitivity Analysis

We performed several sensitivity analyses to assess the robustness of the findings. First, due to the missing information on body mass index in 123,756 (8.7%) women in Sweden and 106,416 (26.4%) women in BC, we performed additional adjustments for BMI based on complete‐case analyses in sensitivity analyses. BMI was calculated using weight measured at registration for antenatal care and self‐reported height. Second, stratified analyses by parity status (nulliparous, 0, or multiparous, ≥ 1) were performed to explore the potential modification of the association between gestational age at delivery and ADHD. Third, we conducted stratified analyses of children's sex to investigate possible differences within these subgroups.

Analyses were performed using SAS version 9.4 (SAS Institute, Cary, North Carolina) and Stata version 17.0. The BC data were analysed in SAS version 9.4. Stata version 17.0 was used to analyse the Swedish data.

### 2.9 Ethics Statement

The study was approved by the Ethics Review Authority in Sweden (approval number 2020‐01545) and the Ethics Committee at the University of British Columbia, Canada (approval number H20‐00902).

## 3 Results

In Sweden, 1,424,453 infants were born to women with low‐risk pregnancies, and in BC, 403,765 infants. Mothers of infants born at 37–38 weeks were more likely to be younger (≤ 19 years old), multiparous with parity ≥ 4, shorter (≤ 159 cm), underweight (BMI < 18.5 kg/m^2^), more likely to have lower educational attainment and a planned caesarean delivery (Table 1). In Sweden, mothers of infants born at 37–38 weeks were more likely to smoke. Notably, infants born at 37–38 weeks in Sweden were more often small for gestational age (< 3^rd^ percentile), while in BC, those born at 41–42 weeks had a higher frequency of being small for gestational age (< 3^rd^ percentile, Table 1). Infants born at 37–38 weeks in both cohorts were more likely to have parents with neurodevelopmental disorders.

**TABLE 1:** Maternal and birth characteristics according to gestational age, singleton term births in Sweden and BC, Canada from 2000 to 2018. [Correction added on 15 February 2025, after first online publication: Tables 1 has been corrected in this version.]

Maternal and birth characteristics	Sweden					British Columbia, Canada				
	Gestational age (weeks)									
	Total	37–38	39–40	41	42	Total	37–38	39–40	41	42
	No. (%)	No. (%)	No. (%)	No. (%)	No. (%)	No. (%)	No. (%)	No. (%)	No. (%)	No. (%)
Total	1,424,453	237,812	790,410	288,651	107,580	403,765	112,563	237,607	48,660	4935
**Maternal age (years)**										
≤ 19	22,148 (1.6)	4308 (1.8)	12,380 (1.6)	3977 (1.4)	1483 (1.4)	6932 (1.7)	2078 (1.9)	3940 (1.7)	823 (1.7)	91 (1.8)
20–24	189,262 (13.3)	32,816 (13.8)	107,093 (13.5)	36,272 (12.6)	13,081 (12.2)	43,660 (10.8)	11,899 (10.6)	25,341 (10.7)	5770 (11.9)	650 (13.2)
25–29	453,841 (31.9)	75,576 (31.8)	254,158 (32.2)	90,692 (31.4)	33,415 (31.1)	101,899 (25.2)	26,941 (23.9)	60,573 (25.5)	13,046 (26.8)	1339 (27.1)
30–34	490,095 (34.4)	79,412 (33.4)	271,455 (34.3)	100,964 (35.0)	38,264 (35.6)	142,773 (35.4)	39,211 (34.8)	84,840 (35.7)	17,017 (35.0)	1705 (34.5)
≥ 35	269,107 (18.9)	45,700 (19.2)	145,324 (18.4)	56,746 (19.7)	21,337 (19.8)	108,501 (26.9)	32,434 (28.8)	62,913 (26.5)	12,004 (24.7)	1150 (23.3)
**Country of birth**										
Nordic	1,110,652 (78.0)	181,027 (76.1)	614,072 (77.7)	230,396 (79.8)	85,157 (79.2)					
Non‐Nordic	312,331 (21.9)	56,561 (23.8)	175,503 (22.2)	57,961 (20.1)	22,306 (20.7)					
Missing	1470 (0.1)	224 (0.1)	835 (0.1)	294 (0.1)	117 (0.1)					
**Country of birth** ^a^										
Canada						255,698 (63.3)	66,493 (59.1)	152,171 (64.0)	33,674 (69.2)	3360 (68.1)
Asia						94,131 (23.3)	31,937 (28.4)	53,231 (22.4)	8128 (16.7)	835 (16.9)
Europe						20,020 (5.0)	4782 (4.3)	12,148 (5.1)	2789 (5.7)	301 (6.1)
Others						15,227 (3.8)	4420 (3.9)	8938 (3.8)	1713 (3.5)	156 (3.2)
Missing						18,689 (4.6)	4931 (4.4)	11,119 (4.7)	2356 (4.8)	283 (5.7)
**Education (years)** ^b^										
≤ 9	116,540 (8.2)	22,083 (9.3)	64,035 (8.1)	21,771 (7.5)	8651 (8.0)					
10–11	150,552 (10.6)	27,139 (11.4)	82,971 (10.5)	29,279 (10.1)	11,163 (10.4)					
12	365,255 (25.6)	61,960 (26.1)	202,986 (25.7)	73,290 (25.4)	27,019 (25.1)					
13–14	203,485 (14.3)	33,695 (14.2)	112,750 (14.3)	41,641 (14.4)	15,399 (14.3)					
≥ 15	577,604 (40.5)	91,102 (38.3)	321,652 (40.7)	120,470 (41.7)	44,380 (41.3)					
Missing	11,017 (0.8)	1833 (0.8)	6016 (0.8)	2200 (0.8)	968 (0.9)					
**Neighborhood income (in quintiles)**										
1st (lowest)						81,361 (20.1)	23,990 (21.3)	46,891 (19.7)	9450 (19.4)	1030 (20.9)
2nd						81,762 (20.3)	23,369 (20.8)	47,587 (20.0)	9821 (20.2)	985 (20.0)
3rd						81,126 (20.1)	22,323 (19.8)	48,023 (20.2)	9846 (20.2)	934 (18.9)
4th						80,574 (20.0)	21,749 (19.3)	48,069 (20.2)	9766 (20.1)	990 (20.1)
5th (highest)						65,579 (16.2)	17,377 (15.4)	39,202 (16.5)	8200 (16.9)	800 (16.2)
Missing						13,363 (3.3)	3755 (3.3)	7835 (3.3)	1577 (3.2)	196 (4.0)
**Year of delivery**										
2000–2004	349,356 (24.5)	60,603 (25.5)	191,388 (24.2)	70,309 (24.4)	27,056 (25.1)	96,236 (23.8)	23,361 (20.8)	56,729 (23.9)	14,414 (29.6)	1732 (35.1)
2005–2009	375,154 (26.3)	65,224 (27.4)	208,207 (26.3)	74,719 (25.9)	27,004 (25.1)	105,879 (26.2)	27,861 (24.8)	62,719 (26.4)	13,958 (28.7)	1341 (27.2)
2010–2014	389,236 (27.3)	63,815 (26.8)	218,509 (27.6)	78,315 (27.1)	28,597 (26.6)	109,916 (27.2)	31,583 (28.1)	65,124 (27.4)	12,032 (24.7)	1177 (23.9)
2015–2018	310,707 (21.8)	48,170 (20.3)	172,306 (21.8)	65,308 (22.6)	24,923 (23.2)	91,734 (22.7)	29,758 (26.4)	53,035 (22.3)	8256 (17.0)	685 (13.9)
**Smoking during pregnancy** ^b^										
Yes	1,267,308 (89.0)	206,552 (86.9)	703,678 (89.0)	259,890 (90.0)	97,188 (90.3)					
No	104,190 (7.3)	20,825 (8.8)	57,252 (7.2)	18,892 (6.5)	7221 (6.7)					
Missing	52,955 (3.7)	10,435 (4.4)	29,480 (3.7)	9869 (3.4)	3171 (2.9)					
**Mothers cohabits with partner**										
Yes	1,273,524 (89.4)	210,422 (88.5)	708,243 (89.6)	258,802 (89.7)	96,057 (89.3)					
No	82,435 (5.8)	14,475 (6.1)	43,929 (5.6)	16,790 (5.8)	7241 (6.7)					
Missing	68,494 (4.8)	12,915 (5.4)	38,238 (4.8)	13,059 (4.5)	4282 (4.0)					
**Marital status** ^a^										
Married						287,478 (71.2)	80,245 (71.3)	169,898 (71.5)	34,041 (70.0)	3294 (66.8)
Divorced/separated/widowed						101,205 (25.1)	28,252 (25.1)	58,864 (24.8)	12,689 (26.1)	1400 (28.4)
Other						15,082 (3.7)	4066 (3.6)	8845 (3.7)	1930 (4.0)	241 (4.9)
**Parity**										
1	683,783 (48.0)	113,879 (47.9)	359,965 (45.5)	145,944 (50.6)	63,995 (59.5)	198,421 (49.1)	51,759 (46.0)	115,935 (48.8)	27,771 (57.1)	2956 (59.9)
2	495,283 (34.8)	82,086 (34.5)	291,449 (36.9)	942,68 (32.7)	27,480 (25.5)	140,495 (34.8)	41,491 (36.9)	83,665 (35.2)	14,075 (28.9)	1264 (25.6)
3	174,405 (12.2)	28,548 (12.0)	99,884 (12.6)	34,898 (12.1)	11,075 (10.3)	45,509 (11.3)	13,485 (12.0)	26,837 (11.3)	4718 (9.7)	469 (9.5)
≥ 4	70,982 (5.0)	13,299 (5.6)	39,112 (4.9)	13,541 (4.7)	5030 (4.7)	19,305 (4.8)	5820 (5.2)	11,144 (4.7)	2096 (4.3)	245 (5.0)
Missing						35 (0.0)	8 (0.0)	26 (0.0)	0 (0.0)	< 5 (‐‐)
**Maternal height (cm)**										
≤ 159	183,567 (12.9)	38,029 (16.0)	102,615 (13.0)	31,149 (10.8)	11,774 (10.9)	68,903 (17.1)	22,925 (20.4)	38,494 (16.2)	6801 (14.0)	683 (13.8)
160–164	355,220 (24.9)	63,214 (26.6)	198,597 (25.1)	68,254 (23.6)	25,155 (23.4)	88,863 (22.0)	25,621 (22.8)	52,091 (21.9)	10,115 (20.8)	1036 (21.0)
165–169	409,010 (28.7)	66,418 (27.9)	227,700 (28.8)	84,019 (29.1)	30,873 (28.7)	84,368 (20.9)	22,377 (19.9)	50,179 (21.1)	10,745 (22.1)	1067 (21.6)
≥ 170	453,299 (31.8)	65,699 (27.6)	248,794 (31.5)	100,705 (34.9)	38,101 (35.4)	86,666 (21.5)	20,114 (17.9)	52,721 (22.2)	12,607 (25.9)	1224 (24.8)
Missing	23,357 (1.6)	4452 (1.9)	12,704 (1.6)	4524 (1.6)	1677 (1.6)	74,965 (18.6)	21,526 (19.1)	44,122 (18.6)	8392 (17.3)	925 (18.7)
**Maternal BMI**										
< 18.5	33,137 (2.3)	7130 (3.0)	19,117 (2.4)	5282 (1.8)	1608 (1.5)	18,845 (4.7)	6010 (5.3)	10,896 (4.6)	1745 (3.6)	194 (3.9)
18.5–24.9	811,731 (57.0)	135,092 (56.8)	458,729 (58.0)	161,189 (55.8)	56,721 (52.7)	191,006 (47.3)	52,348 (46.5)	113,686 (47.9)	22,695 (46.6)	2277 (46.1)
25–29.9	320,361 (22.5)	50,819 (21.4)	173,908 (22.0)	68,257 (23.6)	27,377 (25.4)	58,665 (14.5)	15,650 (13.9)	34,291 (14.4)	7971 (16.4)	753 (15.3)
30–34.9	98,900 (6.9)	16,118 (6.8)	51,970 (6.6)	21,486 (7.4)	9326 (8.7)	19,066 (4.7)	5256 (4.7)	10,907 (4.6)	2649 (5.4)	254 (5.2)
≥ 35	36,568 (2.6)	6018 (2.5)	18,416 (2.3)	8221 (2.8)	3913 (3.6)	9767 (2.4)	2882 (2.6)	5327 (2.2)	1411 (2.9)	147 (3.0)
Missing	123,756 (8.7)	22,635 (9.5)	68,270 (8.6)	24,216 (8.4)	8635 (8.0)	106,416 (26.4)	30,417 (27.0)	62,500 (26.3)	12,189 (25.1)	1310 (26.6)
**Maternal neurodevelopmental disorders**										
Yes	9889 (0.7)	2129 (0.9)	5363 (0.7)	1739 (0.6)	658 (0.6)	6107 (1.5)	1899 (1.7)	3453 (1.5)	682 (1.4)	73 (1.5)
No	1,414,564 (99.3)	235,683 (99.1)	785,047 (99.3)	286,912 (99.4)	106,922 (99.4)	397,658 (98.5)	110,664 (98.3)	234,154 (98.5)	47,978 (98.6)	4862 (98.5)
**Paternal neurodevelopmental disorders**										
Yes	11,066 (0.8)	2056 (0.9)	6022 (0.8)	2156 (0.7)	832 (0.8)	11,263 (2.8)	3266 (2.9)	6602 (2.8)	1268 (2.6)	127 (2.6)
No	1,413,387 (99.2)	235,756 (99.1)	784,388 (99.2)	286,495 (99.3)	106,748 (99.2)	392,502 (97.2)	109,297 (97.1)	231,005 (97.2)	47,392 (97.4)	4808 (97.4)
**Infant sex**										
Male	725,206 (50.9)	118,177 (49.7)	391,380 (49.5)	153,238 (53.1)	62,411 (58.0)	202,788 (50.2)	58,490 (52.0)	117,402 (49.4)	24,453 (50.3)	2443 (49.5)
Female	699,247 (49.1)	119,635 (50.3)	399,030 (50.5)	135,413 (46.9)	45,169 (42.0)	200,977 (49.8)	54,073 (48.0)	120,205 (50.6)	24,207 (49.8)	2492 (50.5)
**Birth weight for gestational age (percentile)**										
< 3	15,223 (1.1)	3329 (1.4)	7407 (0.9)	3082 (1.1)	1405 (1.3)	5499 (1.4)	1055 (0.9)	3403 (1.4)	890 (1.8)	151 (3.1)
3 to < 10	64,801 (4.5)	10,083 (4.2)	3,3659 (4.3)	14,467 (5.0)	6592 (6.1)	19,527 (4.8)	4637 (4.1)	11,788 (5.0)	2691 (5.5)	411 (8.3)
10 to 90	1,185,107 (83.2)	191,859 (80.7)	660,456 (83.6)	242,444 (84.0)	90,348 (84.0)	330,096 (81.8)	92,775 (82.4)	193,892 (81.6)	39,461 (81.1)	3968 (80.4)
> 90 to 97	114,660 (8.0)	21,883 (9.2)	64,360 (8.1)	21,433 (7.4)	6984 (6.5)	34,458 (8.5)	10,145 (9.0)	20,083 (8.4)	3936 (8.1)	294 (6.0)
> 97	42,450 (3.0)	10,240 (4.3)	23,381 (3.0)	6769 (2.3)	2060 (1.9)	14,114 (3.5)	3943 (3.5)	8391 (3.5)	1671 (3.4)	109 (2.2)
Missing	2212 (0.2)	418 (0.2)	1147 (0.1)	456 (0.2)	191 (0.2)	71 (0.0)	8 (0.0)	50 (0.0)	11 (0.0)	< 5 (‐‐)
**Mode of delivery**										
Planned caesarean section	59,120 (4.2)	31,207 (13.1)	24,787 (3.1)	1937 (0.7)	1189 (1.1)	4286 (1.1)	2416 (2.2)	1666 (0.7)	183 (0.4)	21 (0.4)
Emergency caesarean section	87,570 (6.1)	13,142 (5.5)	34,518 (4.4)	22,903 (7.9)	17,007 (15.8)	42,503 (10.5)	9449 (8.4)	24,216 (10.2)	7945 (16.3)	893 (18.1)
Vaginal instrumental	103,329 (7.3)	10,911 (4.6)	51,572 (6.5)	27,592 (9.6)	13,254 (12.3)	51,710 (12.8)	12,326 (11.0)	31,102 (13.1)	7564 (15.5)	718 (14.6)
Vaginal non‐instrumental	1,168,606 (82.0)	180,967 (76.1)	676,665 (85.6)	235,264 (81.5)	75,710 (70.4)	305,266 (75.6)	88,372 (78.5)	180,623 (76.0)	32,968 (67.8)	3303 (66.9)
Missing	5828 (0.4)	1585 (0.7)	2868 (0.4)	955 (0.3)	420 (0.4)					

^a^ Mother's region of birth and marital status were categorized differently in British Columbia, Canada.

^b^ Information on maternal education and smoking was not available in British Columbia, Canada.

During the follow‐up period from 2000 to 2018 in Sweden, 59,989 children were diagnosed with ADHD (rate 4.5 per 1000 child‐years). Correspondingly, in BC, during the same period, there were 27,445 (rate 7.4 per 1000 child‐years) children diagnosed with ADHD.

**FIGURE 1:**
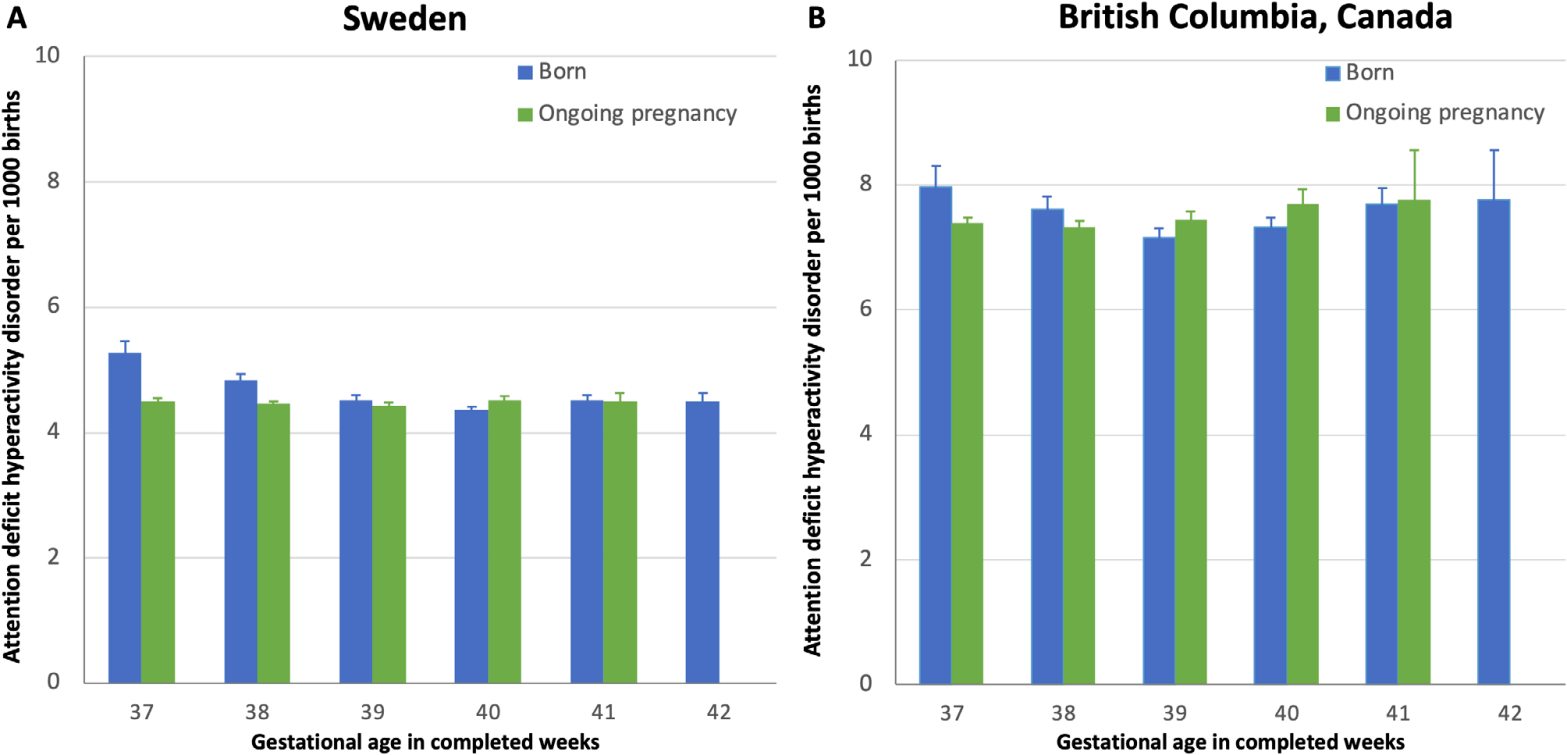
Gestational age and rates of attention deficit hyperactivity disorder in Sweden and BC, Canada, 2000‐2018.

In Sweden, the rate of ADHD was lowest among live births at 40 weeks gestation, and in BC, in those born at 39 weeks, with the highest rates at 37 weeks in both Sweden and BC (Figure 1). In Sweden, the adjusted hazards of ADHD were 10%, 6% and 3%, higher at 37, 38 and 39 weeks gestation compared with those born at ≥ 38, ≥ 39 and ≥ 40 weeks, respectively (Table 2). While in BC, the elevated hazards were at 37 weeks versus ≥ 38 weeks and 38 weeks vs. ≥ 39 weeks (Table 2). In Sweden and BC, the adjusted HR for ADHD was not elevated among infants born at 40 weeks compared with those born at ≥ 41 weeks, with slightly lower rates among those born at 40 weeks.

**TABLE 2:** Gestational age at birth and hazard ratios (RRs) and 95% confidence intervals (CIs) of attention deficit hyperactivity disorder among singleton term births in Sweden and British Columbia Canada. [Correction added on 15 February 2025, after first online publication: Tables 2 has been corrected in this version.]

Gestational age	Deliveries	Follow‐up time, child‐years	No. of cases	Rate, per 1000 child‐years	Unadjusted HR (95% CI)	Adjusted HR (95% CI)^[Link]^, ^b^
**Sweden**						
37 weeks	60,536	610,910	3260	5.34	1.16 (1.12, 1.20)	1.10 (1.06, 1.14)
≥ 38 weeks	1,363,917	13,453,160	61,076	4.54	1.00 (Reference)	1.00 (Reference)
38 weeks	177,276	1,784,938	8708	4.88	1.07 (1.05, 1.10)	1.06 (1.04, 1.09)
≥ 39 weeks	1,186,641	11,668,189	52,368	4.49	1.00 (Reference)	1.00 (Reference)
39 weeks	349,311	3,433,369	15,662	4.56	1.03 (1.01, 1.05)	1.03 (1.01, 1.05)
≥ 40 weeks	837,330	8,234,845	36,706	4.46	1.00 (Reference)	1.00 (Reference)
40 weeks	441,099	4,351,140	19,125	4.40	0.97 (0.95, 0.99)	0.98 (0.96, 1.00)
≥ 40 weeks	396,231	3,883,673	17,581	4.53	1.00 (Reference)	1.00 (Reference)
41 weeks	288,651	2,829,376	12,810	4.53	1.00 (0.97, 1.04)	1.04 (1.00, 1.07)
42 weeks	107,580	1,054,318	4771	4.53	1.00 (Reference)	1.00 (Reference)
**British Columbia, Canada**						
37 weeks	32,796	282,662	2251	7.96	1.09 (1.05, 1.14)	1.09 (1.03, 1.14)
≥ 38 weeks	370,969	3,411,122	25,194	7.39	1.00 (Reference)	1.00 (Reference)
38 weeks	79,767	692,916	5273	7.61	1.05 (1.02, 1.09)	1.08 (1.04, 1.12)
≥ 39 weeks	291,202	2,718,207	19,921	7.33	1.00 (Reference)	1.00 (Reference)
39 weeks	123,196	1,104,913	7906	7.16	0.97 (0.95, 1.00)	1.02 (0.99, 1.05)
≥ 40 weeks	168,006	1,613,294	12,015	7.45	1.00 (Reference)	1.00 (Reference)
40 weeks	114,411	1,076,828	7884	7.32	0.96 (0.93, 1.00)	1.01 (0.96, 1.05)
≥ 40 weeks	53,595	536,467	4131	7.7	1.00 (Reference)	1.00 (Reference)
41 weeks	48,660	484,623	3728	7.69	0.99 (0.89, 1.10)	1.05 (0.93, 1.19)
42 weeks	4935	51,843	403	7.77	1.00 (Reference)	1.00 (Reference)

^a^ Swedish data adjusted for infant's sex, maternal age, country of birth, education, year of delivery, smoking, cohabitation, parity, maternal height, and mother's and father's history of neurodevelopmental disorders. Analysis is based on completed case analysis including 1,326,845 observations. Please see Table S3 for multiple imputations of missing data.

^b^ BC data adjusted for infant's sex, maternal age, country of birth, year of delivery, marital status, parity, maternal height, socioeconomic status, and mother's and father's history of neurodevelopmental disorders. Analysis is based on complete case analysis including 302,723 observation. Please see Table S3 for multiple imputations of missing data.

Table 3 shows the adjusted HRs for ADHD for each country stratified by the onset of delivery. In Sweden, among those with spontaneous onset of labour, 5.2% had an emergency caesarean delivery, 7.3% had an instrumental vaginal delivery and 87.5% had a non‐instrumental vaginal delivery. Among the induced group, 16.2% had an emergency caesarean delivery, 9.9% had an instrumental vaginal delivery and 73.9% had a non‐instrumental vaginal delivery. In BC, following the spontaneous onset of labour, 8.2% had caesarean delivery, 12.4% had an instrumental vaginal delivery and 79.5% had a non‐instrumental vaginal delivery. In the induced group, 19% had an emergency caesarean delivery, 15.4% had an instrumental vaginal delivery and 65.6% had a non‐instrumental vaginal delivery.

**TABLE 3:** Gestational age at birth and rates of ADHD stratified by onset of delivery among singleton term births in Sweden and BC, Canada, 2000–2018.

Onset of delivery: gestational week	Sweden		British Columbia, Canada	
	Number (rate per 1000 child‐years)	Adjusted hazard ratio^a^ (95% CI)	Number (rate per 1000 child‐years)	Adjusted hazard ratio^b^ (95% CI)
Spontaneous labour (weeks)				
37	2218 (5.0)	1.03 (0.99, 1.08)	1688 (7.7)	1.06 (1.00, 1.12)
≥ 38	56,469 (4.5)	1.00 (Reference)	25,021 (7.4)	1.00 (Reference)
38	5481 (4.5)	0.99 (0.96, 1.02)	4269 (7.5)	1.05 (1.01, 1.09)
≥ 39	48,497 (4.5)	1.00 (Reference)	19,821 (7.3)	1.00 (Reference)
39	12,521 (4.4)	1.00 (0.98, 1.02)	6743 (7.0)	1.00 (0.97, 1.04)
≥ 40	34,073 (4.4)	1.00 (Reference)	11,975 (7.4)	1.00 (Reference)
40	16,193 (4.3)	0.96 (0.94, 0.98)	6332 (7.1)	0.98 (0.94, 1.03)
≥ 40	16,385 (4.5)	1.00 (Reference)	4115 (7.7)	1.00 (Reference)
41	10,632 (4.4)	1.01 (0.98, 1.05)	1982 (7.3)	1.01 (0.89, 1.14)
42	4442 (4.5)	1.00 (Reference)	400 (7.7)	1.00 (Reference)
Induced labour (weeks)				
37	495 (7.0)	1.46 (1.34, 1.60)	504 (8.8)	1.17 (1.05, 1.30)
≥ 38	56,469 (4.5)	1.00 (Reference)	25,021 (7.4)	1.00 (Reference)
38	979 (6.6)	1.47 (1.38, 1.57)	862 (8.4)	1.23 (1.14, 1.33)
≥ 39	48,497 (4.5)	1.00 (Reference)	19,821 (7.3)	1.00 (Reference)
39	1080 (5.9)	1.39 (1.30, 1.47)	1040 (7.9)	1.14 (1.06, 1.22)
≥ 40	34,073 (4.4)	1.00 (Reference)	11,975 (7.4)	1.00 (Reference)
40	1281 (5.8)	1.38 (1.30, 1.46)	1497 (8.3)	1.11 (1.04, 1.19)
≥ 40	16,385 (4.5)	1.00 (Reference)	4115 (7.7)	1.00 (Reference)
41	1165 (5.4)	1.32 (1.24, 1.41)	1713 (8.2)	1.11 (0.98, 1.26)
42	4442 (4.5)	1.00 (Reference)	400 (7.7)	1.00 (Reference)
Planned caesarean section (weeks)				
37	225 (6.0)	1.24 (1.09, 1.42)	35 (10.0)	1.57 (1.09, 2.25)
≥ 38	56,469 (4.5)	1.00 (Reference)	25,021 (7.4)	1.00 (Reference)
38	1434 (5.3)	1.19 (1.13, 1.26)	69 (8.0)	1.38 (1.05, 1.80)
≥ 39	48,497 (4.8)	1.00 (Reference)	19,821 (7.3)	1.00 (Reference)
39	747 (4.5)	1.21 (1.13, 1.30)	63 (9.9)	1.35 (0.99, 1.84)
≥ 40	34,073 (4.4)	1.00 (Reference)	11,975 (7.4)	1.00 (Reference)
40	148 (5.1)	1.16 (0.98, 1.36)	31 (12.1)	1.74 (1.15, 2.63)
≥ 40	16,385 (4.5)	1.00 (Reference)	4115 (7.7)	1.00 (Reference)
41	95 (5.3)	1.23 (1.00, 1.50)	20 (16.1)	2.24 (1.27, 3.93)
42	4442 (4.5)	1.00 (Reference)	400 (7.7)	1.00 (Reference)

^**a**^ Adjusted for infant's sex, maternal age, country of birth, education, year of delivery, smoking, cohabitation, parity, maternal height, body mass index and mother's and father's history of neurodevelopmental disorders.

^**b**^ Adjusted for infant's sex, maternal age, country of birth, year of delivery, marital status, parity, maternal height, body mass index, socioeconomic status and mother's and father's history of neurodevelopmental disorders.

In Sweden and BC, infants born after the spontaneous onset of labour at 40 weeks' gestation had no increased HR of ADHD compared with infants born at ≥ 40 weeks (Table 2). In both settings, infants born after the spontaneous onset of labour at 37 weeks had elevated risks of ADHD compared with births at later gestational weeks. Births following induction of labour at any gestational week had higher rates of ADHD compared with births at subsequent gestational weeks (Table 3). In BC, children born following a caesarean delivery at 40 and 41 weeks had the highest rates of ADHD. The lowest adjusted HRs were observed for those born at 38 and 39 weeks compared with those born at a later gestational age. In Sweden, the highest adjusted HR was observed among infants born following a caesarean delivery at 41 weeks. The lowest HR was in infants born at 40 weeks compared with those born at a later gestational week.

### 3.1 Sensitivity Analyses

Our primary results were similar between analyses using complete‐case versus multiple imputations of missing data and adjustment for maternal pre‐pregnancy/early pregnancy BMI (Table S3). The stratified analyses by parity showed similar findings among primiparae and multiparae women in BC; however, in the Swedish cohort, the adjusted hazard ratios for ADHD were not increased at any gestation among births to nulliparous women (Table S4). Lastly, stratified analyses by children's sex produced results consistent with the primary analyses (Table S5).

## 4 Comment

### 4.1 Principal Findings

In this multinational cohort study of low‐risk pregnancies at term gestation in Sweden and BC, we observed increased risks of ADHD among children born at 37, 38 and 39 weeks of gestation when compared with those born at ≥ 38, ≥ 39 and ≥ 40 weeks, respectively. The risk of ADHD was elevated in those born at 41 weeks compared with their counterparts born at 42 weeks. However, the risk of ADHD was lowest for children born at 40 weeks' gestation compared with those born at ≥ 41 weeks, and a slight increase in risk was observed as gestational age at birth increased. This suggests that delivery at 40 weeks is optimal to minimise the risk of ADHD in children who were born as singletons without malformations to mothers with low‐risk pregnancies.

### 4.2 Strengths of the Study

The strengths of our study include linked data from several population‐based registers in two countries. We minimised selection bias by using prospectively collected registry data with minimal losses‐to‐follow‐up. Exposure information of all eligible participants was derived from the register before the diagnosis of the outcomes. Second, the study's large sample size and long follow‐up period provided sufficient statistical power to analyse rare outcomes. Moreover, our categorization of the delivery timing (in gestational weeks) mirrored the obstetric decision‐making setting, allowing us to evaluate the longer‐term prognosis following the induction of labour versus expectant management in low‐risk pregnancies. This information is relevant for clinical decision‐making and pregnancy management.

### 4.3 Limitations of the Data

The study has several limitations that should be considered when interpreting the results. First, although we focused on low‐risk pregnancies, it is possible that some women who underwent induction or caesarean delivery at early‐term gestation had unreported complications that may have affected our findings. This could have led to overestimating the risk of adverse outcomes at 37 and 38 weeks. Second, although we adjusted for the year of delivery in our models, changes in obstetric practices, such as pregnancy dating, may have influenced our findings. Furthermore, using early second‐trimester ultrasonography to ascertain gestational age may have resulted in less precision than using first‐trimester ultrasonography [24], and practices regarding ultrasound dating of gestational age may vary between Sweden and BC. Third, we did not have information on the exact date of induction. However, it is unlikely that the induction and delivery would differ by more than 1 week. However, it may lead to misclassification bias in some cases, for example, when labour is induced at 36 + 6 weeks, but delivery occurs at 37 + 0 weeks, classified as induction at 37 weeks. While relevant, this is a minor limitation in our study. Lastly, we did not account for selection bias or live birth bias in our results, as pregnancies could have ended in stillbirth, infant death or severe disability that would preclude the diagnosis of ADHD, which are severe adverse outcomes that need to be considered during shared decision‐making process about the potential timing of labour induction and caesarean delivery.

### 4.4 Interpretation

The study findings confirm elevated ADHD risks in children born at 37 and 38 weeks' gestation, consistent with previous research on early‐term birth [25, 26, 27]. Despite small effect sizes, the high prevalence of births at 37 and 38 weeks underscores the clinical importance of these results. However, unmeasured confounders may also affect these associations. A recent study suggests a genetic link between gestational age and ADHD, indicating that the genes associated with an increased risk of ADHD may also contribute to a shorter gestational age [28]. Future research should investigate the confounding effects of genetic and environmental factors on the association between time of delivery and long‐term neurodevelopmental outcomes in offspring.

We observed that the risk of ADHD among low‐risk pregnancies was lowest at 39 and 40 weeks of gestation while highest at 37 and 42 weeks. These patterns can be attributed to various factors. Early‐term (37–38 weeks) infants may be vulnerable due to their lower gestational age, while late‐term (41–42 weeks) infants may face risks from conditions associated with prolonged pregnancy, including meconium aspiration syndrome, macrosomia, shoulder dystocia, etc. Brain development accelerates rapidly during the last trimester of pregnancy, reaching 90% of full‐term weight by 38 weeks, potentially explaining the increased vulnerability of early‐term infants [29]. Additionally, pregnancy complications or underlying foetal conditions may prompt early‐term delivery, while declining uteroplacental support in late‐term pregnancies may lead to adverse outcomes for both mother and infant [10, 30]. As gestation advances, there is a substantial decrease in uterine blood flow volume [31], leading to a reduction in growth rate and foetal movement [30]. These physiological changes illustrate the importance of gestational age at delivery, with infants born around 40 weeks having the best health outcomes at birth, which have implications for long‐term neurodevelopment [5, 11, 32, 33, 34].

We found that the gestational age at delivery is associated with ADHD differently in Sweden and BC. In the stratified analyses by the onset of labour, in Sweden, induction at any gestational week was associated with increased ADHD risks, while in BC, there was no statistically significant heightened risk among those born at 41 weeks compared with delivery at 42 weeks. It is essential to consider the variation in clinical practices in obstetric care for low‐risk uncomplicated pregnancies between the two countries: in Sweden, labour induction was offered at 42 + 0 to 42 + 1 weeks [35] and at 42 + 0 weeks after the SWEPIS trial [4], while in BC at 41 + 0 weeks [36]. Notably, in our cohort of low‐risk pregnancies, only 1.2% of women delivered at 42 weeks in BC, whereas in Sweden, the corresponding proportion was 8%. Therefore, labour induction at term gestation in Sweden is more likely due to underlying complications, which can explain some of the variation in results between the two countries. The elevated risk of ADHD observed in children born following induced labour or planned caesarean section may be attributed to these underlying factors. Furthermore, our stratified analyses by parity revealed increased risks of ADHD only in the multiparous group among the Swedish women, with no such elevation observed in the nulliparous group. This might be due to complications in previous pregnancies leading to earlier deliveries.

## 5 Conclusions

In summary, this study suggests that the timing of delivery is associated with a long‐term risk of neurodevelopmental disorders in low‐risk singleton pregnancies at term gestation. Delivery between 37 and 38 weeks is associated with increased risks of ADHD compared with deliveries at a later gestation. In contrast, delivery at 40 weeks is not associated with an increased risk of ADHD. Our findings provide additional prognostic information for clinical practice by informing about infants' long‐term risks and benefits following expectant management of pregnancies beyond 40 weeks' gestation.

## Author Contributions

N.R., T.H.H.N. and Z.H. had full access to all of the data in the study and take full responsibility for the integrity of the data and the accuracy of the data analysis (guarantors of this work). N.R. conceived and designed the study. All authors interpreted the data and critically revised the manuscript for important intellectual content. T.H.H.N. drafted the manuscript and carried out the statistical analysis. N.R. obtained funding.

## Conflicts of Interest

The authors declare no conflicts of interest.

## Supporting information


Data S1.

## Data Availability

Research data are not shared.
