# Supplementary material for: Timing of Delivery of Low‐Risk Persons and the Risk of Attention‐Deficit Hyperactivity Disorder in Offspring: Sweden and British Columbia, Canada
Source: Paediatr Perinat Epidemiol. 2025 Jan 7;39(4):356–69. doi: 10.1111/ppe.13162 (PMC12121333; doi:10.1111/ppe.13162)
Supplement: Supplementary file 1 — Data S1. [file PPE-39-356-s001.docx]

**SUPPLEMENTARY MATERIALS**

**Figure S1**Flow diagram of the study samples, Sweden

**
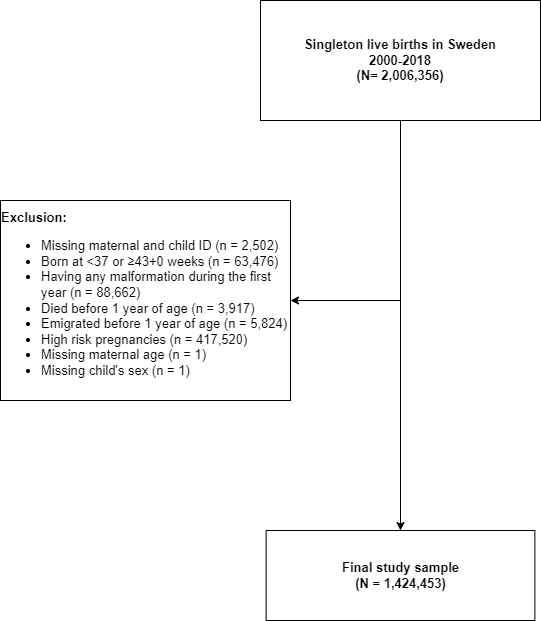
**

**Figure S2**Flow diagram of the study samples, British Columbia, Canada

**
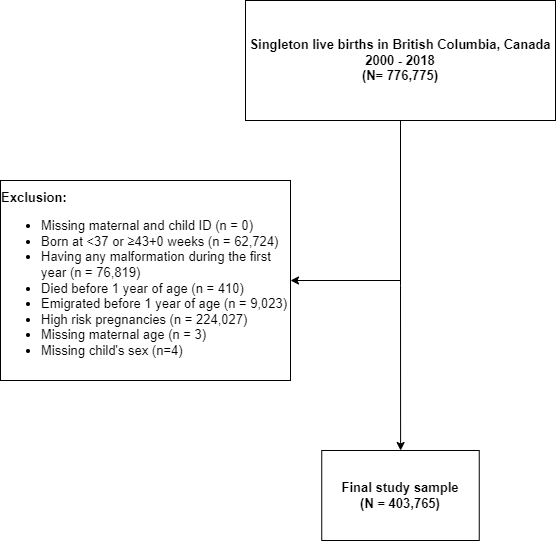
**

**Table S1**. ICD-10 Codes for deriving the Obstetric Comorbidity Index to define high risk pregnancies

| **Co-morbidity** | **ICD-10 Code** |
| --- | --- |
| Alcohol abuse | F10 |
| Asthma | J44, J45 |
| Cardiac Valvular Disease | I05-I09, I34-I39 |
| Chronic Congestive Heart Failure | I50.0 |
| Chronic Ischemic Heart Disease | I20, I25 |
| Chronic Renal Disease | N02.2, N03-N05, N08, N17.1, N17.2, N18, N25 |
| Congenital Heart Disease | Q20-Q26, O99.4 |
| Drug Abuse | F11-F16, F18, F19 |
| Gestational Hypertension | O13, O16 |
| Human Immunodeficiency Virus | B20, B24, O98.7, Z21 |
| Mild/Unspecified Pre-Eclampsia | O11, O14 |
| Placenta Previa | O44 |
| Pre-Existing Diabetes Mellitus | E10, E11, O24.5-O24.7 |
| Pre-Existing Hypertension | I10-I15, O10 and O11+checkbox* |
| Previous Caesarean Delivery | O34.20 +checkbox** |
| Pulmonary Hypertension | I27.0, I27.2, I27.8, I27.9 |
| Pre-Eclampsia | O14, O15 |
| Sickle Cell Disease | D56, D57 |
| Systemic Lupus Erythematosus | M32 |
| *Essential hypertension is also recorded in a checkbox in the prenatal record at first prenatal visit. | |
| **Previous caesarean delivery is also recorded on admission to delivery. | |

**Table S2.** ICD-10 and Anatomical Therapeutic Chemical (ATC) codes for major developmental disorders.

| **Neurodevelopmental disorders** | **ICD-10** | **ATC** |
| --- | --- | --- |
| ADHD# | F90 | N06B |
| **Parental conditions** |  |  |
| Any psychiatric disorders | F00-F99 |  |
| Neurodevelopmental disorders | F84, F70-F73, F78-F79, F90 |  |

ADHD, attention-deficit/ hyperactivity disorder; ICD, international classification of disease;

^#^The ADHD cases were additionally identified through dispensed drugs using the following Anatomical Therapeutic Chemical (ATC) codes, available since 2005 in Sweden and since 1996 in British Columbia: N06BA01 (amphetamine), N06BA02 (dexamfetamine), N06BA04 (methylphenidate), N06BA09 (atomoxetine), N06BA12 (lisdexamfetamine).

| **Table S3. Gestational age at birth and hazard ratios (RRs) and 95% confidence intervals (CIs) of attention deficit hyperactivity disorder among singleton term births in Sweden and British Columbia Canada, adjusted for maternal BMI (Imputed data)** | |
| --- | --- |
| **Gestational age** | **Adjusted HR (95% CI)^a,b^** |
| **Sweden** | |
| 37 weeks | 1.11 (1.07, 1.15) |
| ≥38 weeks | 1.00 (Reference) |
| 38 weeks | 1.07 (1.05, 1.10) |
| ≥39 weeks | 1.00 (Reference) |
| 39 weeks | 1.04 (1.02, 1.06) |
| ≥40 weeks | 1.00 (Reference) |
| 40 weeks | 1.00 (0.98, 1.02) |
| ≥40 weeks | 1.00 (Reference) |
| 41 weeks | 1.05 (1.01, 1.08) |
| 42 weeks | 1.00 (Reference) |
| **British Columbia, Canada** | |
| 37 weeks | 1.08 (1.04-1.13) |
| ≥38 weeks | 1.00 (Reference) |
| 38 weeks | 1.08 (1.05-1.12) |
| ≥39 weeks | 1.00 (Reference) |
| 39 weeks | 1.02 (0.99-1.05) |
| ≥40 weeks | 1.00 (Reference) |
| 40 weeks | 1.02 (0.98-1.06) |
| ≥40 weeks | 1.00 (Reference) |
| 41 weeks | 1.01 (0.91-1.12) |
| 42 weeks | 1.00 (Reference) |
| ^a^Swedish data adjusted for infant’s sex, maternal age, country of birth, education, year of delivery, smoking, cohabitation, parity, maternal height, body mass index, and mother’s and father’s history of neurodevelopmental disorders | |
| ^b^BC data adjusted for infant’s sex, maternal age, country of birth, year of delivery, marital status, parity, maternal height, body mass index, socioeconomic status, and mother’s and father’s history of neurodevelopmental disorders | |

**Table S4. Gestational age at birth and hazard ratios (HRs) and 95% confidence intervals (CIs) for ADHD stratified by parity, in liveborn singleton term infants born in Sweden and BC, Canada**

| **Parity: gestational age** | **Hazard ratio (95%CI)** |  |  |
| --- | --- | --- | --- |
|  | **Sweden*** | **British Columbia, Canada**** |  |
| **Parity = 0** |  |  |  |
| 37 weeks vs ≥38 weeks | 1.04 (0.98, 1.09) | 1.08 (1.01-1.16) |  |
| 38 weeks vs ≥39 weeks | 1.01 (0.98, 1.05) | 1.06 (1.01-1.11) |  |
| 39 weeks vs ≥40 weeks | 1.01 (0.98, 1.04) | 1.04 (1.00-1.09) |  |
| 40 weeks vs ≥41 weeks | 0.97 (0.94, 1.00) | 1.01 (0.95-1.06) |  |
| 41 weeks vs 42 weeks | 1.01 (0.97, 1.06) | 1.06 (0.91-1.23) |  |
| **Parity ≥1** |  |  |  |
| 37 weeks vs ≥38 weeks | 1.17 (1.11, 1.23) | 1.09 (1.01-1.18) |  |
| 38 weeks vs ≥39 weeks | 1.11 (1.08, 1.15) | 1.11 (1.05-1.17) |  |
| 39 weeks vs ≥40 weeks | 1.04 (1.01, 1.07) | 0.99 (0.94-1.04) |  |
| 40 weeks vs ≥41 weeks | 0.99 (0.96, 1.02) | 1.01 (0.94-1.08) |  |
| 41 weeks vs 42 weeks | 1.07 (1.02, 1.13) | 1.05 (0.86-1.29) |  |
| *Adjusted for infant’s sex, maternal age, country of birth, education, year of delivery, smoking, cohabitation, parity, maternal height, body mass index, and mother’s and father’s history of neurodevelopmental disorders | | | |
| **Adjusted for infant’s sex, maternal age, country of birth, year of delivery, marital status, parity, maternal height, body mass index, socioeconomic status, and mother’s and father’s history of neurodevelopmental disorders | | | |

**Table S5. Gestational age at birth and hazard ratios (HRs) and 95% confidence intervals (CIs) for ADHD stratified by sex, in liveborn singleton term infants born in Sweden and BC, Canada**

| **Sex: gestational age** | **Hazard ratio (95%CI)** |  |  |
| --- | --- | --- | --- |
|  | **Sweden*** | **British Columbia, Canada**** |  |
| **Male** |  |  |  |
| 37 weeks vs ≥38 weeks | 1.10 (1.05-1.15) | 1.07 (1.01-1.14) |  |
| 38 weeks vs ≥39 weeks | 1.07 (1.04-1.10) | 1.07 (1.03-1.12) |  |
| 39 weeks vs ≥40 weeks | 1.04 (1.01-1.06) | 1.02 (0.98-1.06) |  |
| 40 weeks vs ≥41 weeks | 0.99 (0.97-1.02) | 1.01 (0.96-1.06) |  |
| 41 weeks vs 42 weeks | 1.05 (1.01-1.10) | 1.07 (0.93-1.24) |  |
| **Female** |  |  |  |
| 37 weeks vs ≥38 weeks | 1.09 (1.02-1.16) | 1.13 (1.02-1.24) |  |
| 38 weeks vs ≥39 weeks | 1.05 (1.01-1.09) | 1.11 (1.03-1.18) |  |
| 39 weeks vs ≥40 weeks | 1.01 (0.98-1.04) | 1.01 (0.95-1.07) |  |
| 40 weeks vs ≥41 weeks | 0.96 (0.93-1.00) | 0.99 (0.91-1.07) |  |
| 41 weeks vs 42 weeks | 1.01 (0.94-1.07) | 1.01 (0.81-1.26) |  |
| *Adjusted for infant’s sex, maternal age, country of birth, education, year of delivery, smoking, cohabitation, parity, maternal height, body mass index, and mother’s and father’s history of neurodevelopmental disorders | | | |
| **Adjusted for infant’s sex, maternal age, country of birth, year of delivery, marital status, parity, maternal height, body mass index, socioeconomic status, and mother’s and father’s history of neurodevelopmental disorders | | | |
